# A Unique Case of Intracranial Mucormycosis Following an Assault

**DOI:** 10.7759/cureus.696

**Published:** 2016-07-17

**Authors:** Fadilah S Hussain, Namath S Hussain

**Affiliations:** 1 Radiology, Wayne State University School of Medicine; 2 Department of Neurosurgery, Loma Linda University Medical Center

**Keywords:** traumatic brain injury (tbi), mucormycosis

## Abstract

Intracranial mucormycosis is a very unusual presentation of an infection after a depressed skull fracture due to an assault. Only sporadic cases have been reported in the literature previously. A 30-year-old male with a traumatic brain injury following an assault, status-post debridement and elevation of a depressed skull fracture, was discharged home several weeks postoperatively. A CT scan of the head with contrast was obtained due to mental status changes and revealed an enhancing ring-shaped lesion in the right frontal lobe consistent with a brain abscess. The patient was taken to the operating room for image-guided excisional biopsy of the lesion, with pathology revealing mucormycosis.

## Introduction

Intracranial mucormycosis is a rare presentation of an uncommon fungal infection after a depressed skull fracture. Only sporadic cases have been reported in the literature previously. Mucormycosis most often afflicts immunocompromised patients, such as those with myelodysplasia [1-3]. However, there have been reports of the disease presenting in patients without any cause for or signs of decreased immune function [4-8]. Diabetes has also been implicated as a contributor to the development of this disease process [2, 9].

## Case presentation

**Figure 1:**
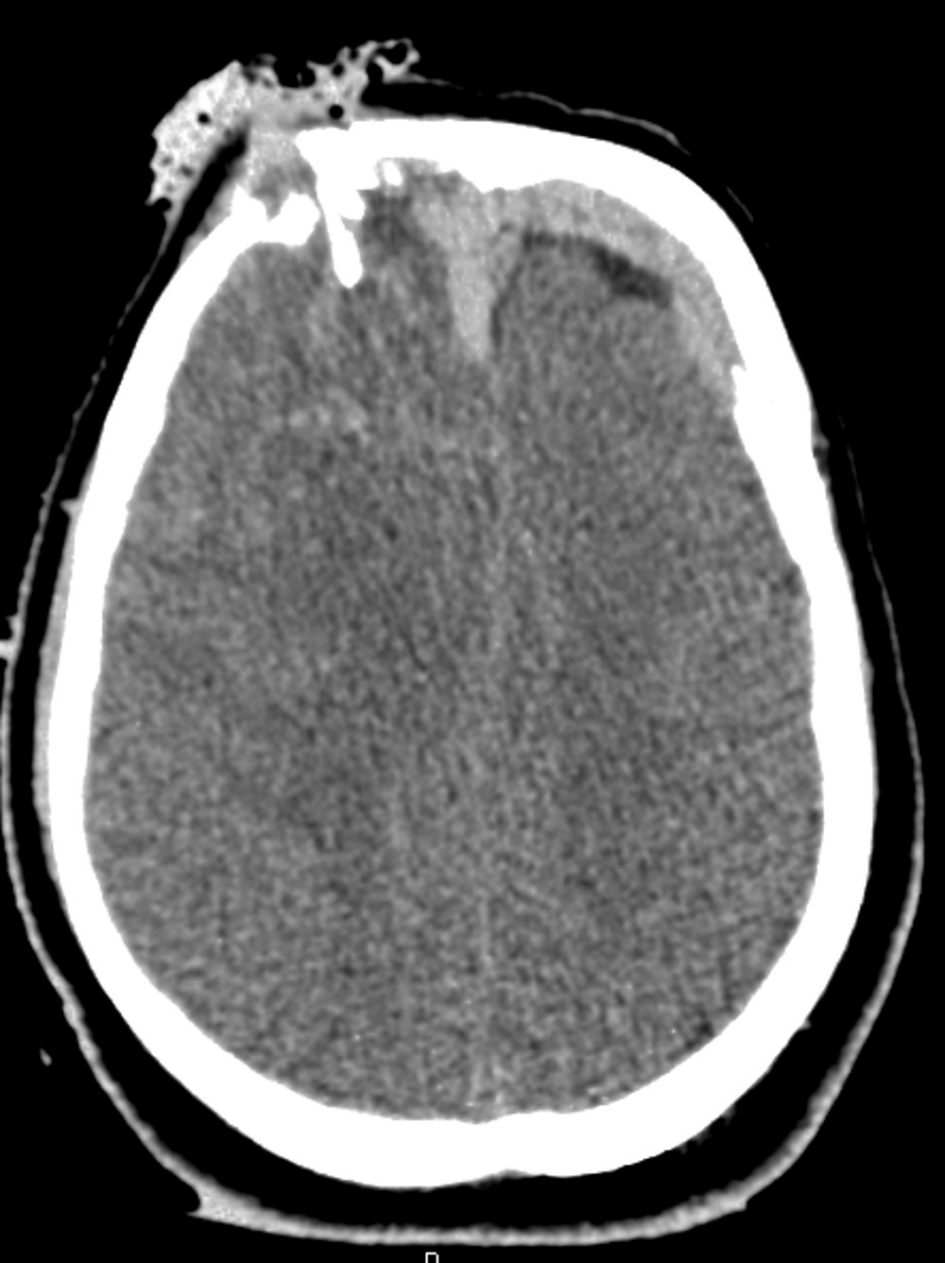
Preoperative CT head showing a depressed skull fracture and associated subdural hematoma

**Figure 2:**
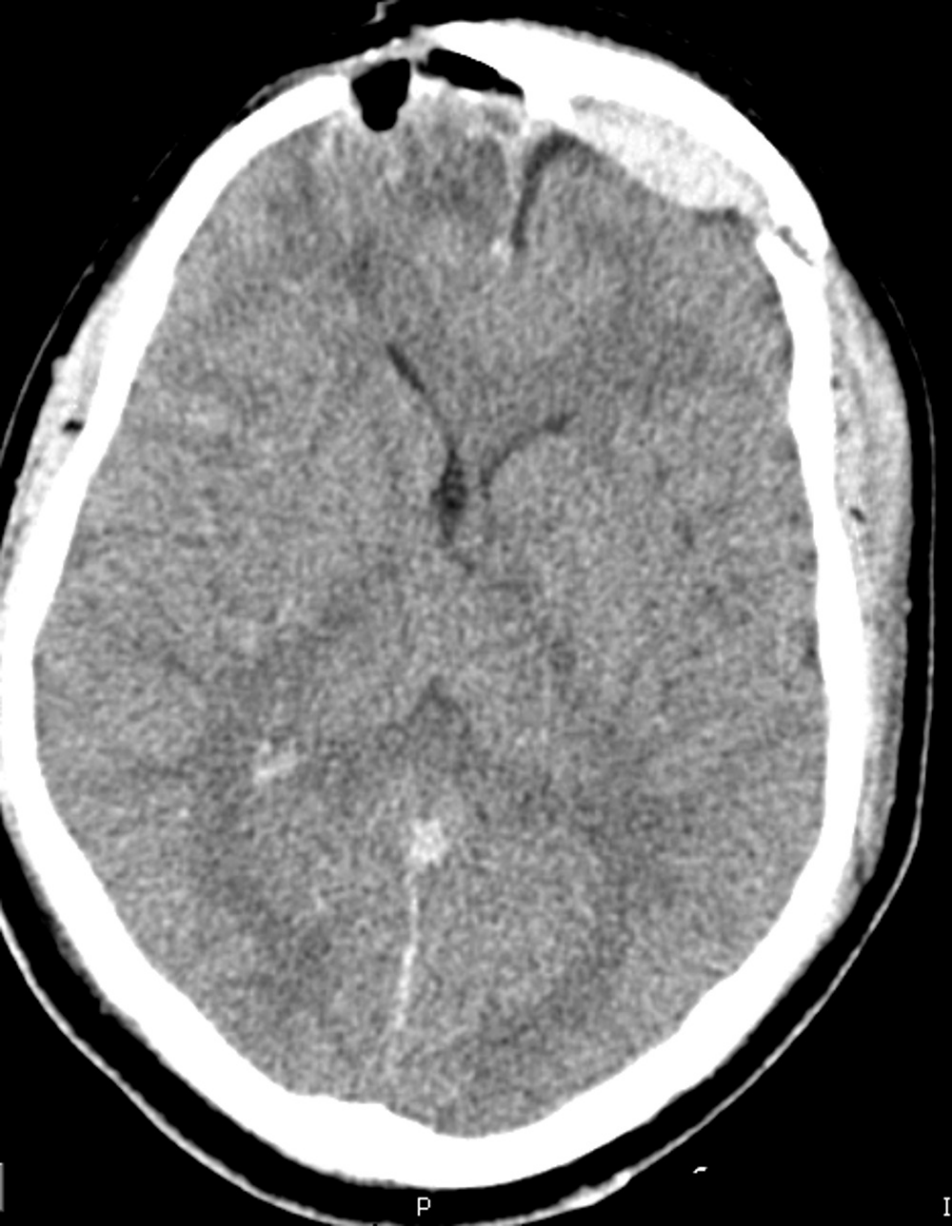
Postoperative CT head showing elevation of fracture fragments

A 30-year-old male with a traumatic brain injury following assault status-post debridement and elevation of a depressed skull fracture was discharged home several weeks postoperatively. His preoperative CT scan is shown in Figure 1, and his postoperative CT shown in Figure 2.

**Figure 3:**
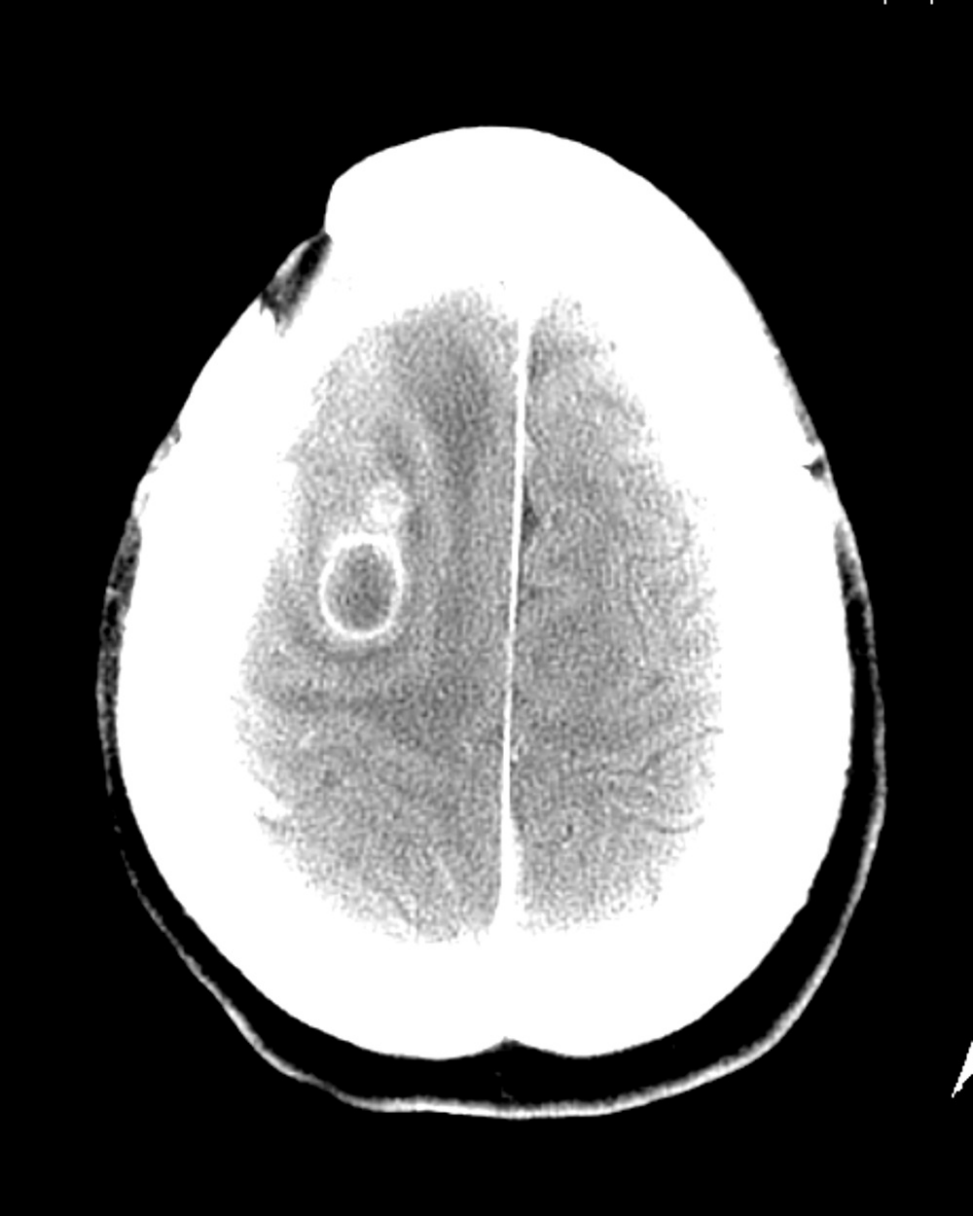
CT head with contrast showing right frontal intraparenchymal brain abscess formation

His mental status at this point stabilized revealing a flat affect, slow verbal responses, and decreased overall cognitive abilities. He had no deficits on gross motor examination. After several weeks of weekly physical therapy and stable cognitive status, he was brought to the hospital again with encephalopathy and confusion. The patient was afebrile and did not have an elevated white blood cell count. He did not have positive blood cultures, consolidation on chest radiograph, a urinary tract infection, or any other typical postoperative or nosocomial infectious process to explain the change in mental status. MRI could not be obtained because of previous eye shrapnel. At this point, a CT scan of the head with contrast was obtained revealing an enhancing ring-shaped lesion in the right frontal lobe consistent with a brain abscess as shown in Figure 3.

The patient was taken to the operating room for image-guided excisional biopsy of the lesion. Surgery was uneventful with gross total resection of the lesion. The patient had no new neurological deficits postoperatively, and his mental status did improve.

**Figure 4:**
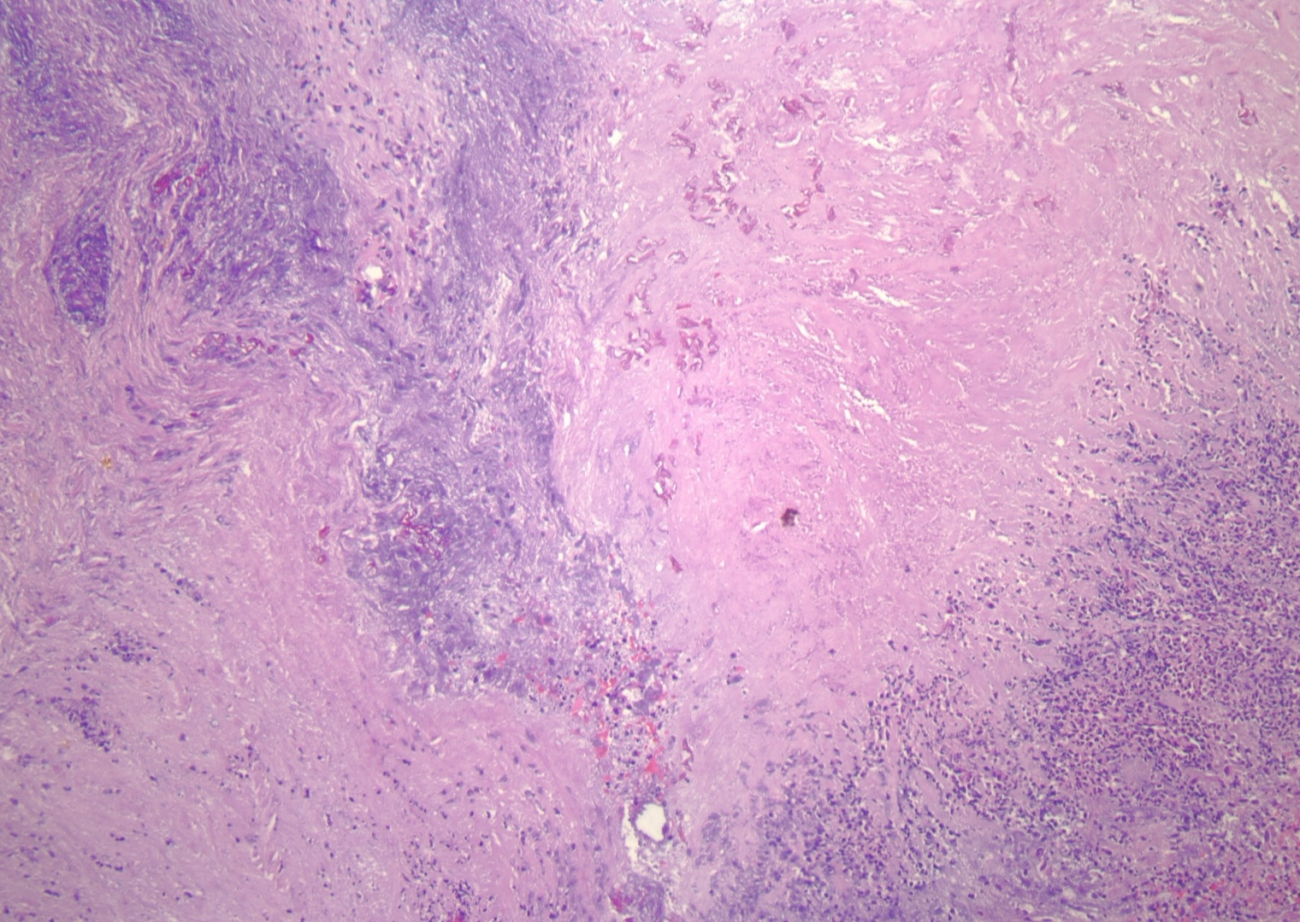
Scanning photomicrograph showing fungal forms surrounded by acute inflammatory cells, fibrin, and necrosis (Hematoxylin and eosin stain; original magnification, X 40)

**Figure 5:**
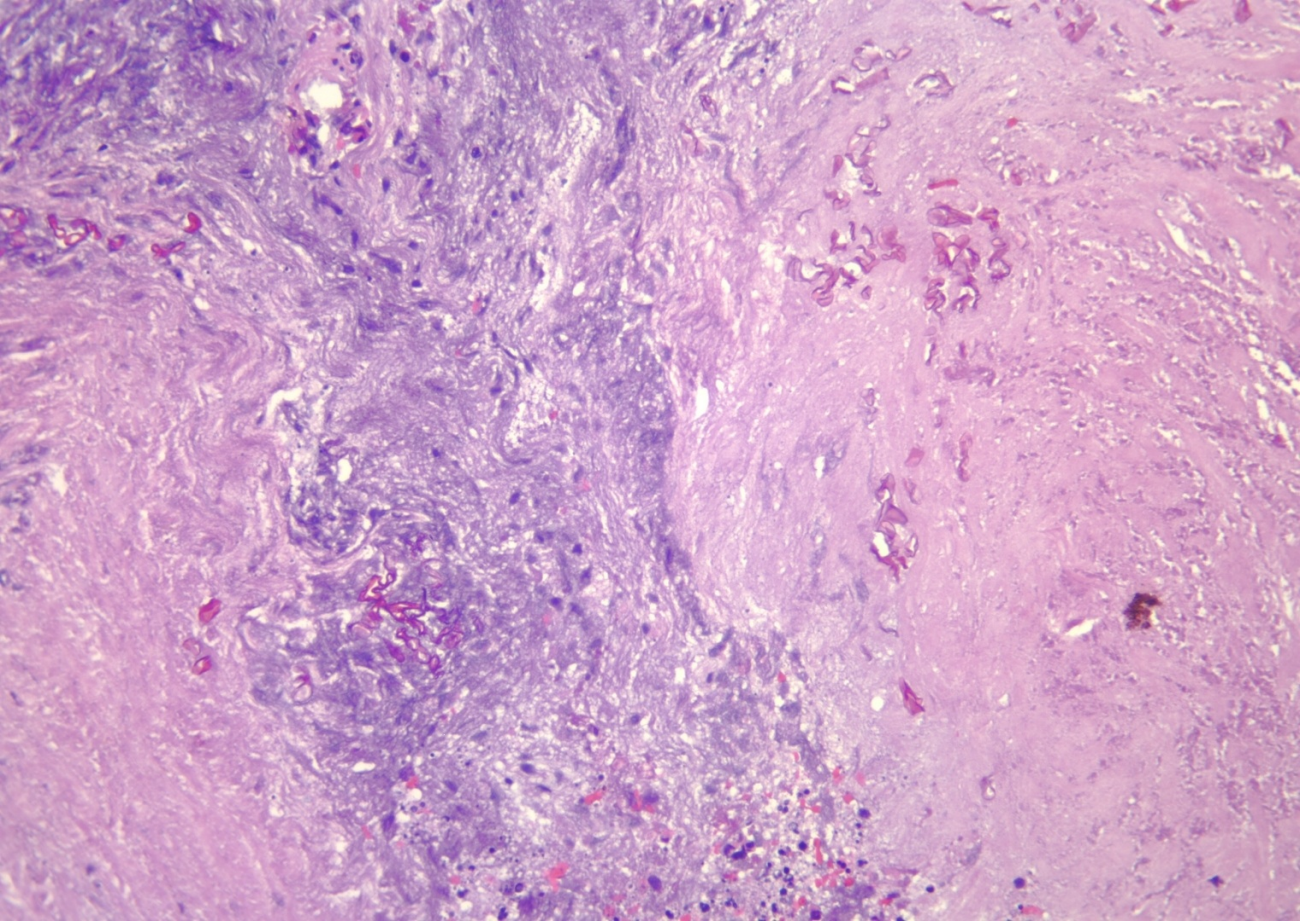
Higher magnification photomicrograph showing fungal hyphae with right angle branching and irregular widths, characteristic of Zygomycetes (Hematoxylin and eosin stain; original magnification, X 100)

**Figure 6:**
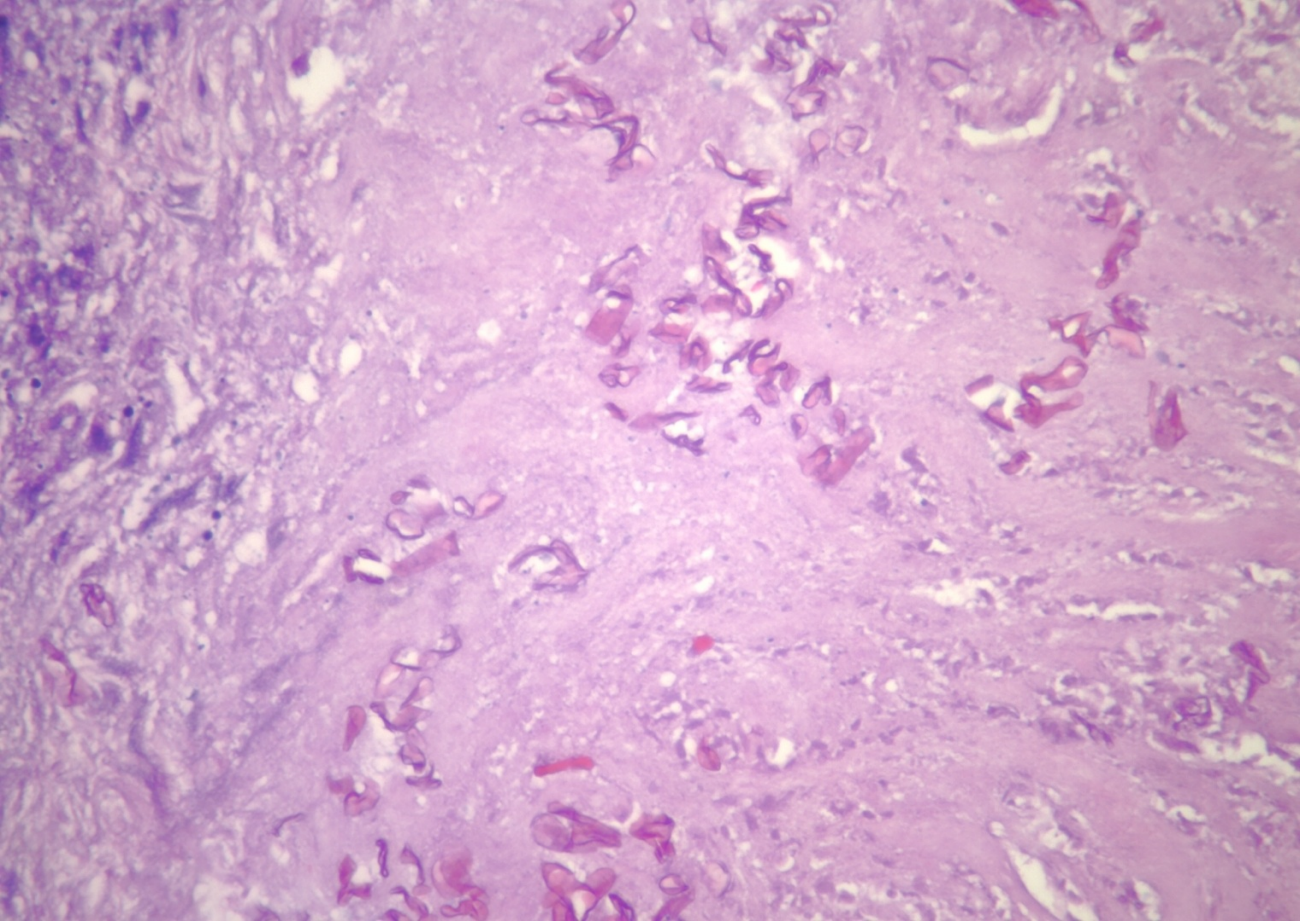
High magnification branching, nonseptate hyphae in a background of necrosis (Hematoxylin and eosin stain; original magnification, X 200)

Pathological examination of the specimen revealed mucormycosis. Specifically, the histology revealed abscess formation with polymorphonuclear cells and necrosis surrounded by granulation tissue and fibrin deposition. Also within the abscess were nonseptate fungal hyphae. The irregular width and right-angle branching pattern are characteristic of zygomycetes. The organisms were readily demonstrated in the areas of necrosis with hematoxylin and eosin staining as shown in Figures 4-6.

**Figure 7:**
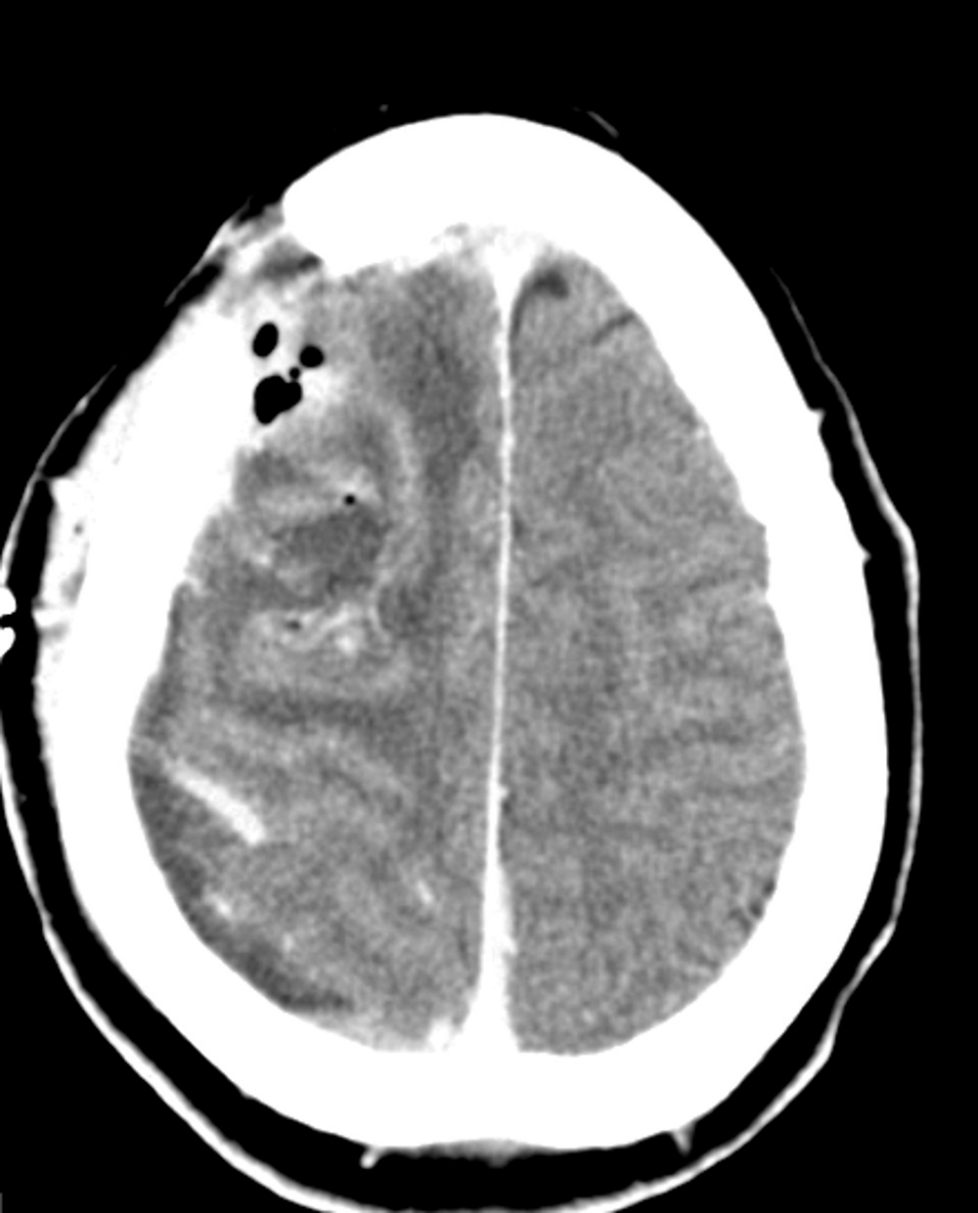
Postoperative CT head with contrast after abscess evacuation

**Figure 8:**
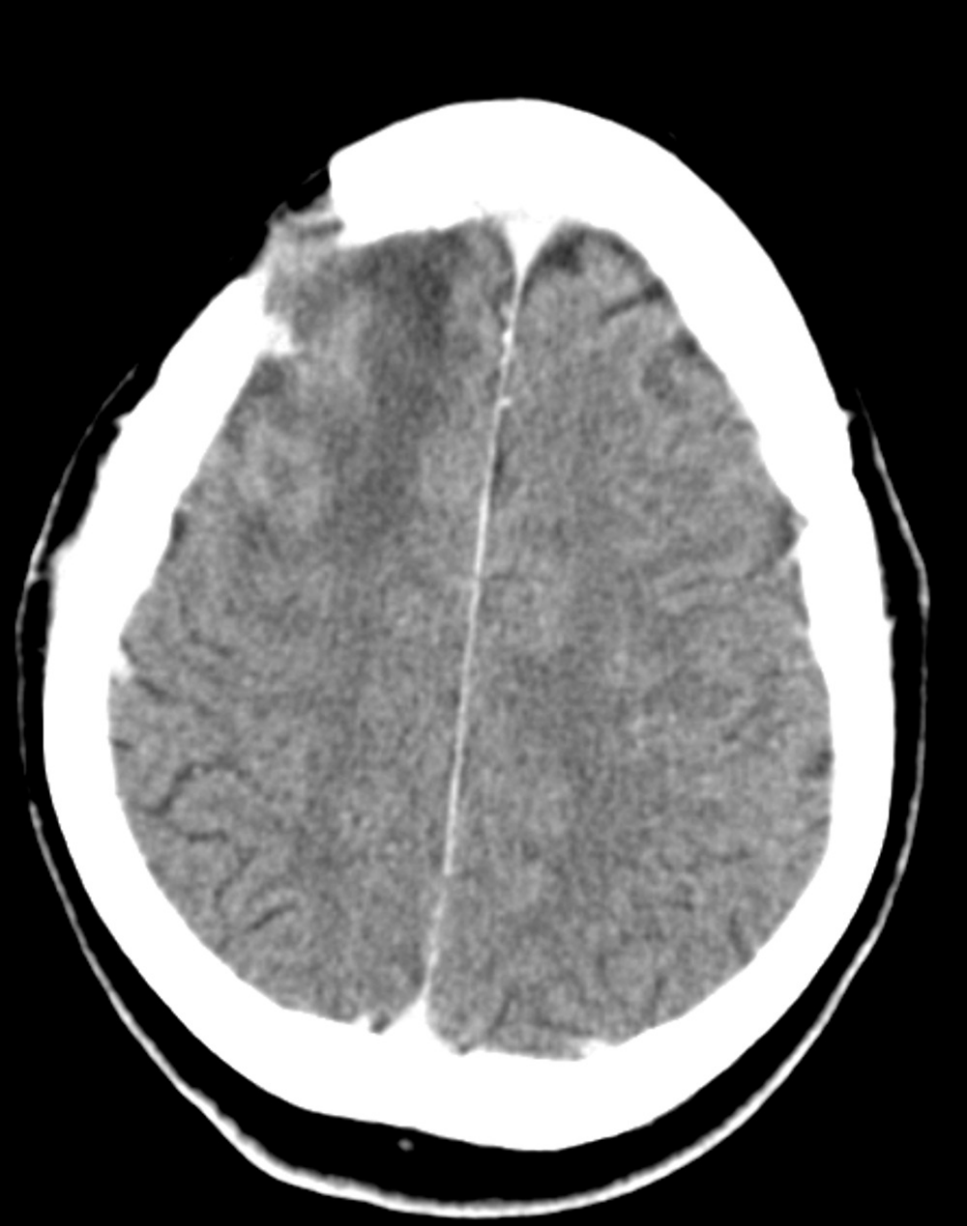
Delayed CT head with contrast showing resolution of abscess

His immediate postoperative CT is shown in Figure 7, and his clinical follow-up CT after amphotericin therapy is shown in Figure 8 showing abscess resolution. 

Informed patient consent for treatment was obtained from the patient's family. No identifying patient information is included in this report.

## Discussion

Our patient had no significant past medical history, was not diabetic, and was not immunocompromised. A PubMed review of the literature revealed only one other report of this type of presentation. Melsom, et al. described a case of a 48-year-old diabetic female who developed a periorbital mucormycosis cellulitis near a deep scalp wound following an assault with a wrench [9]. This infection then progressed to an aggressive necrotizing fasciitis that did not respond to treatment. The patient subsequently developed a hemiparesis, and CT imaging of the head revealed evidence of cavernous sinus invasion of the infectious process which was confirmed on autopsy examination. Also noted was the development of a middle cerebral artery territory infarction.

There have been some reports of systemic mucormycosis associated with cranial injuries. Deja, et al. reported on a patient who developed gastrointestional mucormycosis after a traumatic brain injury [10]. Several reports document the predilection of mucormycosis for invading the facial and paranasal sinuses [1-2, 5, 7], causing an osteomyelitis of the skull base with rare intracranial invasion, termed "rhinocerebral" mucormycosis [1, 7].

## Conclusions

Our patient had no significant past medical history, was not diabetic, and was not immunocompromised. With the rarity of this disease process, it often goes overlooked in the differential diagnosis in patients with new neurological deficits following an assault. CT imaging of the head without contrast will not show this infectious process; thus, it may be missed during the usual workup for mental status changes developing remotely from an assault to the head. Due to the aggressive nature of the disease and the marked improvement that surgery can provide, it is important to keep mucormycosis in the differential diagnosis and to obtain a contrast-enhanced brain imaging study if initial noncontrast imaging is unrevealing.
